# Disparities in COVID-19 vaccine uptake among rural hard-to-reach population and urban high-risk groups of Bangladesh

**DOI:** 10.1371/journal.pone.0302056

**Published:** 2024-04-29

**Authors:** Zerin Jannat, Hemel Das, Md. Wazed Ali, Tasnuva Wahed, Md. Nurul Alam, Md. Jasim Uddin

**Affiliations:** Health Systems and Population Studies Division, icddr,b, Mohakhali, Dhaka, Bangladesh; Jatiya Kabi Kazi Nazrul Islam University, BANGLADESH

## Abstract

**Background:**

Vaccination has been an indispensable step in controlling the coronavirus disease pandemic. In early 2021, Bangladesh launched a mass vaccination campaign to boost the COVID-19 vaccination rate when doses were available and immunized millions in the country. Although deemed a success, disparities became conspicuous in vaccination coverage across population of different socioeconomic background.

**Methods:**

The purpose of this cross-sectional study was to assess the vaccination coverage for three doses and detect disparities in uptake of the COVID-19 vaccine among rural population of hard-to-reach areas and urban individuals belonging to the high-risk group -defined in our study as individuals from elusive population such as floating population/street dwellers, transgender, addicts and disabled population. We conducted household survey (n = 12,298) and survey with high risk group of people (2,520). The collected primary data were analysed using descriptive statistical analysis.

**Results:**

Our findings show that coverage for the first dose of COVID-19 vaccination was high among respondents from both rural Hard-to-reach (HTR) (92.9%) and non-HTR (94.6%) areas. However, the coverage for subsequent doses was observed to reduce significantly, especially for third dose (52.2% and 56.4% for HTR and non-HTR, respectively).

**Conclusion:**

Vaccination coverage among urbanites of high-risk group was found to be critically low. Vaccine hesitancy was also found to be high among individuals of this group. It is essential that the individuals of urban high-risk group be prioritized. Individuals from this group could be provided incentives (transport for disabled, monetary incentive to transgenders; food and medicine for drug user and floating people) and vaccination centers could be established with flexible schedule (morning/afternoon/evening sessions) so that they receive vaccine at their convenient time. Community engagement can be used for both high-risk group and rural population to enhance the COVID-19 vaccination coverage and lower disparities in uptake of the vaccine doses nationwide.

## Introduction

The coronavirus disease (COVID-19) was declared a pandemic by the World Health Organization (WHO) on March 11, 2020 [1]. The pandemic has since wreaked havoc throughout nations and derailed the global economy. Globally, more than 6.9 million deaths from COVID-19 have been reported to the WHO as of November 2023 [2]. The first COVID-19 vaccines became available in 2020 to assuage the dire situation [3]. The vaccines have been credited for reducing the spread, severity and death from the disease. One study claimed that the vaccines have saved 19.8 million lives in 185 countries and nations within a time span of one year [4]. The study also claimed that an additional 600, 000 deaths could have been averted if the 40% full vaccination coverage set by WHO was met by the end of 2021 [4].

The first COVID-19 case in Bangladesh was confirmed on March 8, 2020 and afterwards, the pandemic spread like wildfire, rendering the country the second most infected country in South Asia [5]. The Government of Bangladesh (GoB)) launched a mass vaccination campaign in early 2021 to boost the COVID-19 vaccination rate when doses were available and immunized millions in the country [6]. The GoB targeted to bring 80% of the population, aged 18 years and above, under vaccination [7]. Less than 4% of the country’s population had received two doses of COVID-19 vaccine within early June 2021 but a year later, the number exceeded 67% [8]. However, Bangladesh faces many challenges as it continues its efforts to vaccinate millions more. There are many hard-to-reach (HTR) populations that include the aged and the vulnerable people. Data showed that about 60% of the population aged 60 and above were not vaccinated [9]. Disparity in COVID-19 vaccination was apparent within different age groups, geographical areas, and between different doses (1^st^ and, 2^nd^ doses) [10]. The younger age group had the lowest vaccine coverage in both 1^st^ and 2^nd^ doses compared to other age groups [9]. Data also show that variation in coverage within the divisions such as districts and upazilas of a division with coverage being lowest in Barisal division as of October 2022 [9]. Another prominent challenge observed was the very low coverage of 3^rd^ dose (booster dose). Only 46.2% of 2^nd^ dose recipients received 3^rd^ dose in Bangladesh as of October 2022 [9].

Except vaccine coverage, there might be challenges in vaccine acceptability side. Vaccine hesitancy has been defined by the SAGE Working Group as delay in accepting or refusing the vaccination despite availability of vaccination services [11]. Notably, vaccine hesitancy had been declared as one of the top ten global health threats by WHO in 2019 [12]. In Bangladesh, one study measured and found prevalence of COVID-19 vaccine hesitancy to be high, i.e. 46.2% during the early 2021 [13]. Studies also identified that gender (female, transgender), low family income, married people, tobacco users, administrative divisions, country of origin, religion, individuals who did not experience physical illnesses in past year, people with opposite political affiliations, vaccination knowledge, negative attitude and beliefs on conspiracy toward COVID -19 vaccine, perceived benefit and barriers about vaccine, individuals doubtful about vaccine efficacy, little to no concern about getting infected, COVID-19 infection history were more likely to be vaccine hesitant [13–15]. Evidence also showed lack of demand for vaccine and vaccine hesitancy to be significantly associated with lower vaccine acceptance [16].

To ensure increased equity in COVID-19 vaccination uptake, it is essential to study coverage of COVID-19 doses among the vulnerable of Bangladesh. It is also momentous to document disparities and explore challenges in regards to receiving COVID-19 vaccine, among rural population who reside in HTR and non-HTR areas and the urban high-risk group. Considering such need, this study assessed, captured and explored disparities in COVID-19 vaccine uptakes among different vulnerable groups, their reasons as well as enablers and disablers behind the rural and high- risk group individuals for getting or not getting vaccinated. This study also explored the existing challenges and overcoming them in regards to uptake of COVID-19 vaccination. Findings of this study confirm disparities in COVID-19 vaccination equity among the vulnerable individuals of Bangladesh. If the need arises today or tomorrow, these findings will help policymakers to design mass campaigns, that can enable bringing a greater number of the Bangladeshi individuals, especially the marginalized, under vaccine coverage.

## Methods

### Study area

Bangladesh has 8 divisions that branch out into 64 districts. These districts further divide into 495 upazilas (i.e. sub-districts) and 12 city corporations (CCs) [17]. We conducted this study from December 14, 2022 to February 28, 2023, in purposively selected 16 districts and three city corporations (CCs) of Bangladesh. The areas were selected based on low vaccination coverage as of March 2022, obtained from COVID-19 Vaccination Dashboard for Bangladesh [9]. The study was conducted in randomly selected upazilas (i.e. sub-districts) and wards from high-risk 16 districts and three CCs of Bangladesh. To elaborate, we collected the complete list of upazilas and administrative wards from our 16 districts and 3 CCs, respectively from Bangladesh National Portal [17]. We randomly selected four upazilas from each district of all 16 districts and five wards from each CC for the study.

### Study population

The study population included rural population residing in HTR and non-HTR areas and urbanites of the high-risk group, all aged 18 years and above. Notably, ‘high-risk group’ in our study encompassed individuals from elusive population such as floating population/street dwellers, transgender, addicts and disabled population. We selected our rural and high-risk urban individuals as our study population to be on par with our objective to detect and address disparities in coverage of COVID-19 vaccine doses among the vulnerable population of Bangladesh.

### Ethics approval and consent to participate

The Research Review Committee (RRC) and Ethical Review Committee (ERC) of Institutional Review Board (IRB) of International Centre for Diarrhoeal Disease Research, Bangladesh (icddr,b) approved this study prior to data collection and implementation. Furthermore, respondents were interviewed after obtaining their written informed consent. Participation was voluntary and the participants were ensured that their refusal would have no adverse consequences for them. All information shared by participants remain confidential, under lock and key and only investigators of this study have access to the shared/ collected information. Any personal identifiable information was processed under secured conditions with access to limited appropriate staff, i.e. investigators and authors of this study.

### Data collection

Data for this study was collected using pre-tested household survey at rural areas and survey of high-risk groups in CCs. To develop the questionnaire, we first considered and then selected indicators most relevant to our study objectives. We pre-tested the questionnaire by sending the data collectors to a field test and assess their data collection before sending them to study areas. Notably, we used the same questionnaire with same sets of question but entitled them differently and according to the study population and area. To elaborate, we entitled the survey for rural HTR and non-HTR residents as ‘Household Survey” and the survey for high-risk group individuals as ‘Surveys of high-risk groups in CCs’.

We recruited 57 filed staff for data collection. Among them, 38 were field research assistants 19 field supervisors. They were divided into teams and conducted data collection at 19 sites of 16 districts and 3 CCs. They obtained written informed consent from respondents first and the collected data via in-person interview using the questionnaire. The data collection period was from December 14, 2022 to January 31, 2023 and the study wrapped up on February 28, 2023.

### Sampling and sample size for household survey in rural HTR and non-HTR areas

Household survey was conducted with the household head or spouse. Respondents were asked to provide information on their socio-demographic characteristics and COVID-19 vaccination status of all adult members of the household. The response rate at rural areas was approximately 97%.

Data were collected on COVID-19 vaccination status, COVID-19 infection (confirmed/symptomatic), perceptions related to the COVID-19 infection and vaccination, reasons for accepting/not accepting vaccine and hesitancy of receiving vaccine. Notably, the vaccination status was confirmed by checking the vaccine card first. If, however, the vaccine card was unavailable, we confirmed the status by enquiring about their vaccination history verbally. Data quality was ensured through random uninformed spot-checks and 5% re-interview by supervisors and checking consistency across variables during data cleaning.

The 2^nd^ dose coverage of COVID-19 vaccine in selected 16 districts stood at 66.4% as on 17 October 2022 [9]. Three-stage (upazilas within a district, clusters within upazila and households within cluster) random cluster sampling procedure was considered for calculation of the sample size per district. Hence, the minimum required sample size was 763 eligible people per district for estimating 66.4% coverage of the 2^nd^ dose COVID-19 vaccine (4), with 95% confidence interval, 5% points margin of error, 10% non-response rate and design effect 2. The required sample size was 763 (rounded to 768) per district, amounting to a total of 12,288 from 16 districts (Table 1). Thus, the formula we used for calculation of the sample size is:

$$n=\frac{{Z_{\alpha }}^{2}P(1-P)}{d^{2}}\times design\;effect\times factor\;to\;adjust\;for\;non‐response\;rate$$

where P = 2^nd^ dose coverage of COVID-19 vaccine in selected 16 districts,

Z_α_ = 1.96 at α = 0.05 and d = margin of error, the design effect = 2 and factor to adjust for a non-response rate of 10%.

**Table 1:** Three-stage cluster sampling and sample size calculation.

Stage 1 sample units per district	Stage 2 EAs per 4 upazilas	Stage 3 households per EA	Required sample per district	Total Districts	Total required sample
4 upazilas	32	24	768	16 districts	12,288

Notably, not all the villages are considered to be HTR in an HTR upazila. Some people live in less/non-HTR areas and may have better access to required services than those residing in actual HTR areas. To estimate differences in vaccination coverage and hesitancy between HTR and non-HTR areas, we selected 75% of the required clusters/enumeration areas (Eas) from HTR clusters and 25% from non-HTR clusters from the selected upazilas.

We randomly selected 32 clusters (24 HRT and 8 non-HRT) using probability proportional to population size (PPS) sampling procedure from randomly selected four upazilas per selected district. Total number of clusters in 16 districts was 512 (= 32 cluster × 16) for this study.

### Selection process

The Bangladesh Bureau of Statistics (BBS) created enumeration areas (Eas) around 120 households at each mouza/village in the entire country for field operation of the National Housing and Population Census 2022. The EPI out-reach centers (each had a well-defined catchment area, termed as EPI cluster) are classified as HTR and non-HTR clusters. The selection process followed different steps and details are as below:

Step 1: The EAs of sampled upazilas were matched with EPI out-reach clusters to divide the EAs into HTR and non-HTR EAs. The list of HTR EAs and non-HTR EAs was form of the sampling frames of HTR EAs and non-HTR EAs for random selection of 24 HTR EAs and 8 non-HTR EAs for the survey. To do this, data collectors collected the list of EPI centers from the upazila’s EPI micro-plan and then bifurcated centers under the list of HTR and non-HTR. Using random sampling, catchment areas of 6 EPI centers from HTR and 2 EPI from non-HTR areas were selected. At the catchment area of each EPI center, two data collectors collected data from 24 households. Thus, 192 (32x6) interviews were conducted from each upazila.

Step 2: Our team visited selected households to obtain consent of the household head or spouse for the interview. According to BDHS 2017–18, the mean size of household was 4.3 and percentage of 18 years and above population equalled 61.5 (7). Hence, it was expected that a household would have approximately 3 (~4.3×61.5%) eligible individuals for COVID-19 vaccination.

Step 3: Household head (or wife in his absence) was the respondent who reported on behalf of household members aged 18 years and above.

#### Sampling and sample size for high-risk groups in CCs

Unlike rural areas, there is no list of high-risk groups (floating population/street dwellers, transgenders, addicts, disables) at the CCs. Following discussions with city dwellers, ward councillors (local public representatives), social welfare officers and NGO workers, we purposively selected five wards with maximum number of high-risk people per CC to capture more people in different high-risk groups. Since such individuals gather at late night around different public points (such as mazar, foot over bridge, railway stations, bus stations, launch/boat stations, large shopping mall etc) for sleep, a list of public points per ward was prepared for planning survey schedule. High-risk group members at each point was visited during early morning and at night to prevent repetition. The data collectors searched for individuals who belonged to the high-risk groups within a kilometre of the area selected from each selected ward. The response rate was approximately 90%. Data quality was ensured through spot-checking, 5% re-interview by supervisors and checking consistency across variables during data cleaning.

### Analyses of household survey data

The COVID-19 vaccination coverage, which is the outcome variables, was calculated for household members by considering the vaccination information from both vaccination card and history. Notably, information on vaccination of all eligible household members were provided by respondents of the survey.

Foremostly, the analysis of percentage distribution of background characteristics; coverage of COVID-19 vaccination; reasons behind vaccination hesitancy were explored. The distribution of sample characteristics investigated through profile analysis by location of household for both male and female participants was done. We used sex ratio to compare the distribution of male and female in the sample by district. While inspecting information related to COVID-19 vaccine, place of vaccination and reasons of vaccine hesitancy was analysed. Furthermore, COVID-19 vaccination coverage by intake of doses by background characteristics of respondents was also examined by bivariate analysis. We also conducted two sample proportion tests among the categories of each variable where we kept one category as a reference group for analysing vaccination coverage. The variables that showed significant effect on vaccination doses in two sample proportion tests were considered as potential independent variables in the regression model. Binary Logistic regression model was applied to the dataset to explore the adjusted effect of several covariates on COVID-19 vaccine doses adjusting for clustering at EPI outreach center level.

### Analyses of high-risk group survey data

The main focus during analysis of data for high-risk group was to obtain information regarding COVID-19 vaccination coverage and hence the vaccination coverage percentage of each of the three doses by several socio demographic characteristics such as age, gender, occupation, income quantile, perceived health status were analysed through bivariate analysis. We performed binary logistic regression model considering the respondents with complete information for all characteristics. This was done to observe the adjusted effect of covariates that were found to be significant in two-sample proportion test on the vaccine doses, Binary logistic regression model was performed considering the respondents with complete information for all characteristics.

## Results

We conducted a total of 12,298 interviews for household survey at rural areas and 2,520 with high-risk group at CCs. Results of our surveys are presented below:

### Socio-demographic characteristics

#### Socio-demographic characteristics of the respondents

S1 Table depicts socio demographic characteristics of respondents from the surveys we conducted. The age of respondents varied from 18 years to exceeding 55 years–with respondents somewhat evenly distributed among different age groups. Most of our respondents were women and homemakers– 66.2% and 59.0%, respectively. Approximately, one-fourth of the household respondents had no education and were poor (i.e. second quintile). A high number of the respondents were deemed to be healthy with only 10% being afflicted with chronic illness. Less than 1% of the respondents were disabled. Among the high-risk group in CCs, most were aged above 55 years and males. A high number of respondents, i.e. 72.4%, had no institutional education. Approximately, 70% of the respondents were beggars and one-third of the respondents earned an income within the range of BDT 3000—BDT 5999. Most respondents, i.e. 69.6%, were healthy whereas more than 15% suffering from chronic illnesses and 14% of the respondents were disabled. Notably, respondents from the high-risk group had greater number of individuals who were disabled or suffered from chronic illnesses compared to respondents of the other survey.

#### Gender distribution of household members

The total number of household members at the rural areas equalled 30,283 among which 13,812 and 16,471 were males and females, respectively (Table 2). Approximately, 75% of the males and females resided in hard-to reach areas. The sex ratio of rural population was 0.84 indicating a greater number of females residing at the rural areas. At the urban areas, the total number of respondents equalled to 2,520 and among them, 1,720 and 742 were males and females, respectively. About 58 individuals were Transgender. Notably, the sex ratio of urban population was 2.3 indicating a greater number of males were interviewed in urban areas.

**Table 2:** COVID-19 vaccination coverage by district.

District	# of household interviewed	Sample			Doses (%)								
					1^st^			2^nd^			3^rd^		
		Male	Female	Total	Male	Female	Total	Male	Female	Total	Male	Female	Total
Bandarban	768	820	947	1,767	83.4	77.2	80.1	78.5	73.3	75.7	32.1	31.0	31.5
Rangamati	773	1,038	1,111	2,149	90.3	88.3	89.3	82.9	81.4	82.1	39.0	41.0	40.0
Lakshmipur	768	765	950	1,715	86.9	81.1	83.7	74.9	66.3	70.1	36.1	30.4	32.9
Shariatpur	769	757	950	1,707	91.1	91.5	91.3	83.9	86.3	85.2	49.9	56.0	53.3
Madaripur	768	660	938	1,598	78.2	71.3	74.2	65.8	60.7	62.8	44.2	40.7	42.2
Rajbari	768	985	1,098	2,083	75.0	79.1	77.2	68.1	75.0	71.7	40.9	49.8	45.6
Faridpur	768	767	964	1,731	91.5	91.9	91.7	84.7	85.8	85.3	47.2	51.9	49.8
Gaibandha	768	934	1,082	2,016	90.0	90.9	90.5	81.5	84.8	83.2	41.9	46.5	44.3
Kurigram	768	875	992	1,867	87.3	90.6	89.1	76.9	83.9	80.6	41.5	48.8	45.4
Jhalokathi	768	759	950	1,709	85.0	89.6	87.5	83.4	87.6	85.7	55.9	59.2	57.7
Pirojpur	768	912	1,124	2,036	92.2	92.0	92.1	87.2	88.2	87.7	50.8	54.3	52.7
Barisal	768	864	1,073	1,937	86.6	89.6	88.2	82.1	84.0	83.1	48.4	50.0	49.3
Bhola	768	881	1,019	1,900	93.3	90.0	91.5	87.5	85.1	86.2	45.4	44.7	45.1
Netrokona	771	916	1,059	1,975	94.7	94.8	94.7	89.0	90.7	89.9	57.4	63.9	60.9
Mymensingh	768	903	1,017	1,920	92.4	89.6	90.9	84.4	83.5	83.9	44.4	48.5	46.6
Sunamganj	769	976	1,197	2,173	92.4	93.4	93.0	87.6	88.6	88.2	56.3	56.7	56.5
**Total (Rural: Household survey)**	**12,298**	**13,812**	**16,471**	**30,283**	**88.3**	**87.7**	**88.0**	**81.4**	**81.8**	**81.6**	**45.7**	**48.6**	**47.3**

### COVID-19 vaccination coverage

Table 2 shows the district wise COVID-19 vaccination coverage in accordance to gender and vaccine doses. Coverage is somewhat even for males and females across the 1^st^, 2^nd^ and 3^rd^ doses of COVID-19 vaccine at the districts, i.e. rural areas. More than 80% of both male and female respondents from these areas had taken the 1^st^ and 2^nd^ doses of the vaccine. However, less than 50% of the household members from both categories of genders had opted to take the 3^rd^ dose. The highest number of household members who took 1^st^ and 2^nd^ doses of COVID-19 vaccine resided in Netrokona district. The highest number of male and female who took 3^rd^ dose resided in Sunamganj and Jhalokathi districts, respectively among household members of all the 16 selected districts. Notably, the coverages of first and second doses were worse in Madaripur (1^st^ dose: 74.2%, 2^nd^: 62.8%, 3^rd^: 42.2%) and Rajbari (1^st^ dose: 77.2%, 2^nd^: 71.7%, 3^rd^:45.6%) compared to other districts. Data also revealed that 3^rd^ dose vaccination coverage was quite low in Bandarban (31.5%) and Lakshmipur (32.9%) districts.

**Fig 1:**
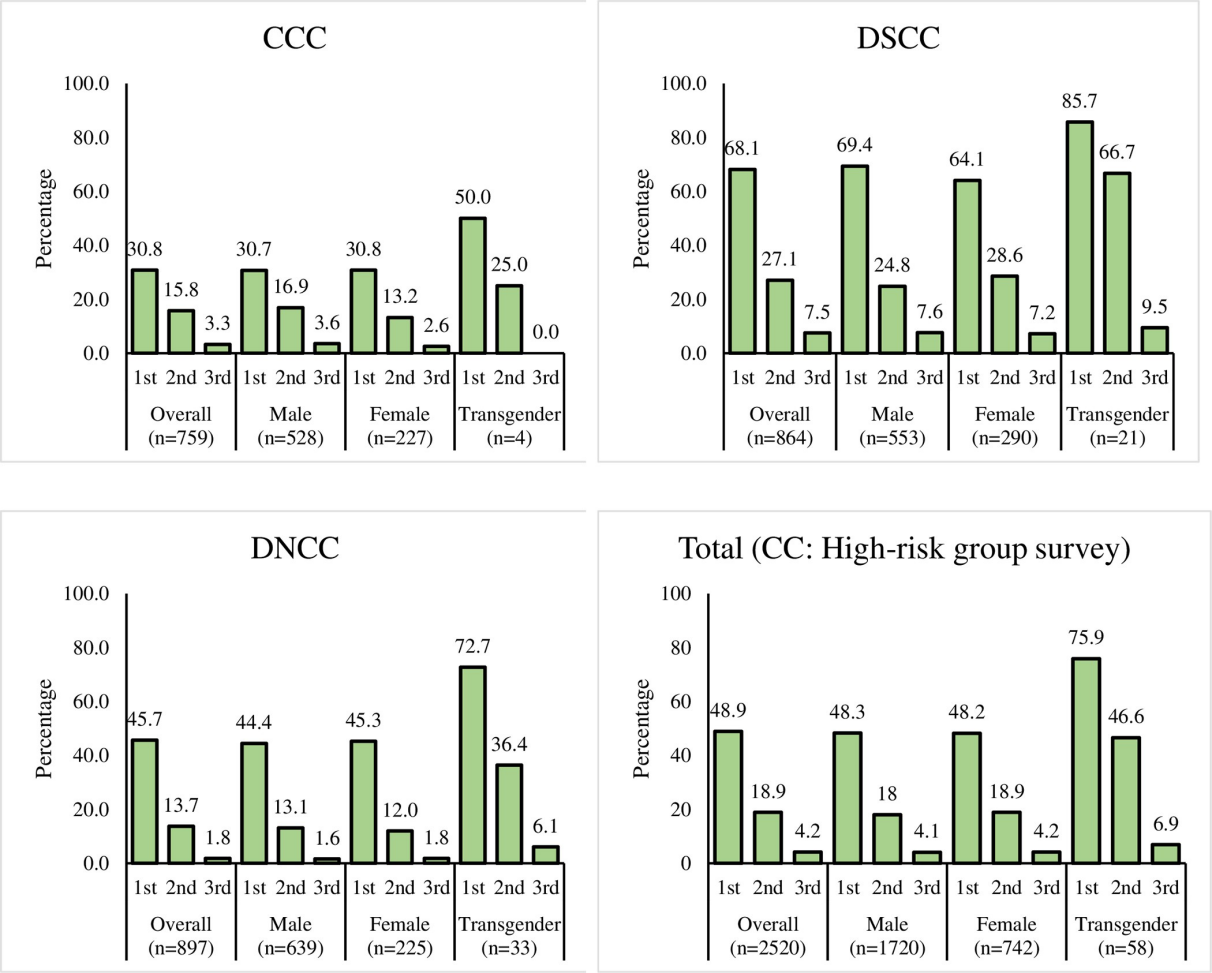
COVID-19 vaccination coverage by CC.

COVID-19 vaccine coverage among respondents of urban areas (high risk group) was much lower compared to respondents of the districts/rural areas (Fig 1). Less than 50% of urban males and females received 1^st^ dose of vaccines. Across the doses, lesser respondents opted to take the vaccine–less than 20% of both genders received the 2^nd^ dose of vaccine whereas less than 5% chose to get vaccinated with the 3^rd^ dose. However, it is to be noted that Trans individuals residing at the CCs accepted the highest number of all doses compared to urban males and females. About 76% and 47% of the Trans individuals had accepted 1^st^ and 2^nd^ doses of COVID-19, respectively. We observed from household and high-risk group surveys that the highest percent of respondents had received all their vaccine doses at a vaccination campaign followed by upazila health complex (S2 Table).

### Disparities in vaccine coverage

Table 3 depicts COVID-19 vaccination coverage among respondents by dose, survey location and socio-demographic characteristics. Here, we compared the difference in prevalence of vaccination coverage according to several characteristics of respondents with a reference group and its significance was assessed by two sample proportion tests.

**Table 3:** COVID-19 vaccination coverage by dose and socio-demographic characteristics of the respondents in rural and city corporation areas.

Characteristics	Survey location							
	Rural: Household survey (n = 12,298)				CC: High-risk group survey (n = 2,520)			
	Total	Dose (%)			Total	Dose (%)		
		1^st^	2^nd^	3^rd^		1^st^	2^nd^	3^rd^
**Household location**								
Hard-to-reach (ref.)	9,274	92.9	87.5	52.2	-	-	-	-
Non-hard-to-reach	3,024	94.6**	88.9**	56.4***	-	-	-	-
**Age**								
18–29 (ref)	3,115	88.3	78.7	38.3	401	54.1	18.5	3.5
30–39	3,468	95.7***	91.0***	56.4***	474	57.4	21.7	4.6
40–54	3,491	96.6***	93.1***	61.1***	677	48.7*	20.2	4.0
55+	2,224	91.6***	87.5***	57.0***	968	42.7***	16.8	4.4
**Gender**								
Male (ref)	4,151	94.2	88.4	52.4	1720	48.3	18.0	4.1
Female	8,147	93.0**	87.6	53.7	742	48.2	18.9	4.2
Transgender	-	-	-	-	58	75.9***	46.6***	6.9
**Education status**								
No education (ref.)	3,121	93.2	88.4	54.3	1824	46.9	17.3	3.5
Primary incomplete	2,065	92.5	87.1	51.8**	506	52	20	5.1**
Primary completed	2,070	92.7	86.5**	51.2**	120	60.8**	30***	10***
Secondary incomplete	2,588	93.4	87.6	52.8	49	51	28.6**	6.1
Secondary completed or higher	2,454	94.8**	89.2	55.4	21	76.2**	52.4***	9.5
**Occupation**								
Household chores (ref.^1^)	7,260	93.2	87.6	54.2	-	-	-	-
Farmer	1,882	95.2**	90.8***	51.4**	-	-	-	-
Business	764	95.9**	89.4	53.8	-	-	-	-
Day labourer	924	92.9	86.2	48.8**	61	68.9***	42.6***	9.8***
Student	352	98.0***	91.8**	38.6***	-	-	-	-
Service	437	95.4*	91.5**	63.6***	-	-	-	-
Beggar	-	-	-	-	1711	50.3***	20.7***	4.6**
Not working^2^/ Disabled (ref.^3^)	595	84.2***	78.2***	54.8	563	34.6	9.4	2
Porter	-	-	-	-	170	72.4***	21.2***	5.3**
Others^4^	84	85.7**	78.2**	54.8	15	71.4**	50.0***	7.1
**Wealth quintiles**								
Lowest (ref.)	2,467	91.0	86.1	46.5	-	-	-	-
Second	3,146	93.4***	88.0**	51.4***	-	-	-	-
Middle	2,166	94.8***	89.7***	55.2***	-	-	-	-
Fourth	2,853	93.7***	86.7	56.5***	-	-	-	-
Highest	1,666	94.5***	89.8***	58.7***	-	-	-	-
**Income quartiles**								
Less than Tk. 3000	-	-	-	-	530	32.8	6.2	0.4
Tk. 3000—Tk. 5999	-	-	-	-	836	40.6**	14.5***	2.9**
Tk. 6000—Tk. 8999	-	-	-	-	735	59***	23.8***	5.3***
Greater or equal Tk. 9000	-	-	-	-	408	68.9***	35.8***	9.8***
**Perceive health condition**								
Healthy (ref.)	10,979	94.0	88.4	53.1	1755	54.8	21.3	4.6
Chronic illness	1,273	88.3***	83.5***	54.9	411	29.7***	9,0***	2.4**
Disabled	46	78.3***	67.4***	41.3	354	42.1***	18.6	4.5
**Total**	**12,298**	**93.4**	**87.8**	**53.3**	**2,520**	**48.9**	**18.9**	**4.2**

^1^ Reference for household survey.

^2^ For high-risk group it is coded as vagabond.

^3^ Reference for high-risk group survey.

^4^Others: Driver, Rickshaw/van puller, Skilled worker, Retired person, old-age allowance.

* p < 0.1

** p < 0.05

*** p < 0.01(compared to the reference category.

The household survey, conducted in rural areas, shows that the coverage of 1^st^ dose was above 90% among respondents from both HTR and non-HTR areas. This figure, however, reduced for the subsequent number of doses for respondents of both HTR and non-HTR areas. The difference in vaccination coverage by household location is statistically significant at 5% level of significance. Coverage of each vaccine dose and decline in subsequent doses were evenly distributed among respondents of various age groups, gender, educational status, occupation and wealth status. The COVID-19 vaccination coverage differed significantly among age-groups compared to respondents aged 18–29 years for all subsequent doses. High significant difference in COVID-19 vaccination coverage for all doses was also found for different level of wealth quintiles considering the lowest wealth quintile as referent group. A similar scenario was observed for individuals who were in different states of health condition although the healthy individuals opted the most to get vaccinated whereas the disabled respondents opted the least overtime with increasing number of doses and overall.

The high-risk group survey, conducted in the CCs, depict some interesting results. The percent of high-risk respondents who received 1^st^ doses of COVID-19 vaccine was almost half than that of the respondents from household survey (i.e. rural areas). It was observed that a greater number of younger respondents, i.e. aged from 18–39 years, accepted the vaccine doses. In regards to gender, it was the Transgender group who accepted the vaccine doses most, almost twice than the male and female respondents we interviewed–i.e. approximately, 76% of the Transgenders had taken the 1^st^ dose of COVID-19 vaccine compared to 48% of the males and 48% of the females who accepted this dose. Notably, the percentage of male and female respondents from high-risk group was substantially low, i.e. less than 20% and 5% for the 2^nd^ and 3^rd^ doses of vaccine, respectively. In regards to education, figures show that those with higher level of education were much more likely to get vaccinated. To exemplify, 76.2% and 52.4% of the respondents with secondary completed or higher education took the 1^st^ and 2^nd^ doses of vaccine, in contrast to 46.9% and 17.3% of the respondents who had no education, respectively. Porters, vagabonds, day labourer and beggars were the highest among those who accepted the 1^st^ dose of the vaccine but these percentages declined over the subsequent number of doses. Furthermore, those who had a monthly income of BDT 9,000 and above was the highest group to accept the vaccine. The difference in vaccination coverage among different categories of income quintile was highly significant for all doses compared to respondents with income less than 3000 taka. Lastly, in regards to health condition, healthy respondents were the ones to take the vaccine doses most but a vice versa scenario was observed for the respondents suffering from chronic illnesses. Notably, across the groups, respondents from high-risk group had low significantly low vaccination coverage (irrespective of their health condition) when compared to respondents from rural areas (i.e. household survey).

We also enquired vaccine card availability and expenses incurred among respondents who had received at least one dose of the COVID-19 vaccine by survey type (S3 and S4 Tables). It was found from the household survey, 77% of the respondents mentioned that their vaccine cards were available. The vice versa was observed for the respondents of high-risk group survey–approximately 92% of these respondents did not have their vaccine card with them. In regards to expenses, a high number of respondents from the household and high-risk group survey had spent on getting registered for vaccine/vaccine card– 95.8% and 82.4%, respectively with less than 10% reporting that they had spent of other types of expenses.

### Reasons for receiving and not receiving COVID-19 vaccine

In this section, the enabling factors for receiving multiple doses among the participants by different types of surveys are presented.

S5 Table shows the reasons that motivated respondents who received at least a dose of COVID-19 vaccine, in accordance to both surveys. We accepted multiple responses for this enquiry from the respondents. Protecting oneself from the virulent COVID-19 was the biggest (90.4% for the household survey and 80.4% in high-risk group survey) motivation for the respondents across all surveys to accept vaccine doses. In the household survey, protecting their family was also another prominent reason for accepting COVID-19 vaccine. In the high-risk group, respondents mentioned that they followed others in regards to accepting the COVID-19 vaccine.

Table 4 presents the reasons mentioned by the respondents for not receiving doses, of COVID-19 vaccine. We accepted multiple responses for this enquiry from the respondents. Most respondents from the household survey, i.e. 32%, mentioned illness as the cause for not getting vaccinated with 1^st^ dose. Approximately 25% of the respondents mentioned that they had neglected to getting vaccinated with the 2^nd^ and 3^rd^ doses. From the high-risk group survey, most respondents opted not getting the 1^st^ dose of COVID-19 vaccine due to lack of faith on the vaccine. Furthermore, one-fourth of the respondents mentioned registration difficulties as another reason for not accepting the 1^st^ dose of vaccine. In regards to the 2^nd^ and 3^rd^ doses, most of them mentioned that they did not take the subsequent doses out of negligence.

**Table 4:** Reasons for not getting vaccinated by survey type and vaccine doses.

Reasons for not receiving vaccine*	Survey type (%)					
	Rural: Household survey			CC: High-risk group survey		
	1^st^ dose	2^nd^ dose	3^rd^ dose	1^st^ dose	2^nd^ dose	3^rd^ dose
	(n = 3,087)	(n = 1,887)	(n = 10,081)	(n = 1,288)	(n = 724)	(n = 370)
Due to the illness	32.0	16.3	13.0	20.1	5.7	8.3
Due to pregnancy	18.0	9.9	2.9	3.5	0.6	0.3
Fear of side-effects	14.5	10.8	10.3	17.2	2.9	4.1
Neglected	13.6	24.4	25.2	14.4	24.0	27.6
Does not have faith on vaccination	11.2	2.4	0.7	28.7	3.0	1.1
Was busy	7.2	15.2	15.4	4.5	6.5	14.6
Registration difficulties	5.9	5.2	2.5	25.8	1.1	1.9
Fear of needles	5.7	1.5	0.9	3.8	0.7	0.8
Thinking of getting vaccinated later	5.4	11.7	13.2	5.9	7.9	20.3
Was not at home	4.8	10.2	9.7	0.5	1.2	3.0
Don’t know	4.5	4.1	4.7	0.8	3.2	2.2
Inconvenient time	2.6	4.5	4.1	3.2	4.4	5.7
Vaccination center is located in a distant location	2.4	5.3	2.7	3.3	3.0	1.9
Change of residence	1.2	1.6	1.6	0.9	7.9	14.3
Didn’t know that the vaccine should be taken	0.7	2.0	4.3	10.3	32.3	13.5
Did not know where to go for vaccination	0.6	2.1	2.8	14.8	31.1	15.4
Did not have sufficient money	0.2	0.9	0.3	0.7	0.1	1.1
Others	3.7	3.9	3.8	5.1	14.5	3.0

*Multiple responses.

Table 5 shows results of the multiple logistic regression model. From the rural household survey, it was observed that rural residents of non-HTR areas were more likely to accept the different doses of the COVID-19 vaccine. To exemplify, non HTR respondents were 1.3 times likely than the HTR to receive the first does of the vaccine. Respondents older in age, were found to be more likely to accept the vaccine doses. This is especially conspicuous among the respondents aged 40–54 years who were 5.4, 5.1, and 3 times likely to accept the 1^st^, 2^nd^ and 3^rd^ doses of COVID-19 vaccine. Similarly, individuals with greater level of education, especially those who had completed secondary or higher level of education were 1.6, 1.7, and 1.5 times more likely to accept all the consecutive three doses of the vaccine compared to those with no education. Furthermore, the better the financial state of a respondent, the more likely were they to get vaccinated. This is depicted by asset quintile with all the quintiles being positive and statistically significant compared to the lowest quintile. However, we found that respondents suffering from chronic diseases and those who had disability, were less likely to accept the consecutive doses of COVID-19 vaccine compared to healthy respondents.

**Table 5:** Multiple logistic regression model for household survey and survey of vulnerable people by doses.

Characteristics	Survey location					
	Rural: Household survey			CC: High-risk group survey		
	Adjusted OR (95% CI)^1^			Adjusted OR (95% CI)		
	1^st^	2^nd^	3^rd^	1^st^	2^nd^	3^rd^
**Household location**						
Hard-to-reach (ref.)	1	1	1	-	-	-
Non-hard-to-reach	1.3 (1.0–1.7)*	1.1 (0.9–1.4)	1.1 (1.0–1.3)	-	-	-
**Age (years)**						
18–29 (ref)	1	1	1	1	1	1
30–39	3.6 (2.9–4.5)***	3.3 (2.8–3.9)***	2.3 (2.0–2.5)***	1.3 (1.0–1.8)*	1.4 (1.0–2.0)*	1.5 (0.8–3.1)
40–54	5.4 (4.2–6.8)***	5.1 (4.3–6.1)***	3.0 (2.7–3.4)***	1.2 (0.9–1.6)	1.8 (1.3–2.5)***	1.9 (0.9–3.7)*
55+	2.9 (2.3–3.7)***	3.6 (2.9–4.4)***	2.8 (2.4–3.3)***	1.2 (0.9–1.6)	1.9 (1.3–2.7)***	2.9 (1.4–5.7)***
**Gender**						
Male (ref)	1	1	1	1	1	1
Female	0.8 (0.6–1.1)	1.1 (0.9–1.5)	1.1 (0.9–1.3)	0.9 (0.7–1.1)	1.0 (0.8–1.2)	1.0 (0.6–1.5)
Transgender	-	-	-	2.0 (1.1–3.8)**	2.8 (1.5–5.0)***	1.4 (0.5–4.3)
**Education status**						
No education (ref.)	1	1	1	1	1	1
Primary incomplete	0.9 (0.7–1.2)	1.0 (0.8–1.2)	0.9 (0.9–1.1)	0.9 (0.7–1.1)	0.9 (0.7–1.3)	1.2 (0.8–2.0)
Primary completed	1.0 (0.8–1.3)	1.1 (0.9–1.3)	1.0 (0.9–1.2)	1.2 (0.8–1.8)	1.6 (1.1–2.5)**	2.6 (1.3–5.2)***
Secondary incomplete	1.3 (1.0–1.7)**	1.4 (1.2–1.7)***	1.2 (1.0–1.3)***	0.9 (0.5–1.9)	1.5 (0.7–2.9)	1.4 (0.4–4.8)
Secondary completed or higher	1.6 (1.2–2.2)***	1.7 (1.4–2.2)***	1.5 (1.3–1.7)***	3.6 (1.2–11.0)**	6.1 (2.1–17.6)***	3.5 (0.7–16.8)
**Occupation**						
Household chores (ref.^1^)	1	1	1	-	-	-
Farming	1.1 (0.7–1.6)	1.3 (0.9–1.9)	0.8 (0.7–1.0)**	-	-	-
Business	1.1 (0.7–1.8)	1.0 (0.7–1.4)	0.8 (0.7–1.0)*	-	-	-
Day-labour	0.7 (0.5–1.1)	0.9 (0.7–1.3)	0.8 (0.7–1.0)	2.4 (1.3–4.3)***	3.6 (1.9–6.7)***	2.1 (0.7–6.3)
Studentship	4.8 (2.2–10.4)***	2.7 (1.7–4.1)***	0.8 (0.6–1.0)*	-	-	-
Service	0.9 (0.5–1.5)	1.2 (0.8–1.8)	1.1 (0.9–1.4)	-	-	-
Begging	-	-	-	1.9 (1.6–2.4)***	2.0 (1.5–2.9)***	1.6 (0.8–3.2)
Not working^2^/	0.4 (0.3–0.6)***	0.6 (0.4–0.8)***	0.9 (0.7–1.1)	1	1	1
Disabled (ref.^3^)						
Porter	-	-	-	2.7 (1.8–4.0)***	1.3 (0.8–2.2)	1.2 (0.5–3.1)
Others^4^	0.4 (0.2–0.8)***	0.5 (0.3–0.8)***	0.9 (0.6–1.4)	2.2 (0.7–7.3)	3.7 (1.2–11.0)**	0.9 (0.1–8.1)
**Asset quintiles**						
Q1 (ref.)	1	1	1	-	-	-
Q2	1.4 (1.1–1.8)***	1.2 (1.0–1.4)*	1.2 (1.0–1.4)**	-	-	-
Q3	1.7 (1.2–2.2)***	1.3 (1.1–1.6)**	1.4 (1.2–1.6)***	-	-	-
Q4	1.3 (1.0–1.6)**	1.0 (0.8–1.2)	1.4 (1.2–1.6)***	-	-	-
Q5	1.3 (1.0–1.7)**	1.2 (0.9–1.5)	1.4 (1.2–1.7)***	-	-	-
**Income quartiles**						
Less than BDT 3,000	-	-	-	1	1	1
BDT. 3,000–5,999	-	-	-	1.3 (1.0–1.7)**	2.4 (1.6–3.6)***	7.8 (1.8–33.2)***
BDT6,000–8,999	-	-	-	2.5 (2.0–3.3)***	4.5 (3.0–6.8)***	15.7 (3.7–66.3)***
Greater or equal to BDT 9,000	-	-	-	3.1 (2.3–4.2)***	7.3 (4.7–11.3)***	29.5 (6.9–126.5)***
**Perceive health condition**						
Healthy (ref.)	1	1	1	1	1	1
Chronic illness	0.4(0.3–0.5)***	0.5 (0.4–0.7)***	0.9 (0.7–1.0)**	0.4 (0.3–0.5)***	0.4 (0.3–0.6)***	0.6 (0.3–1.3)
Disabled	0.3 (0.1–0.8)***	0.3 (0.2–0.7)***	0.7 (0.4–1.2)	0.6 (0.5–0.8)***	0.9 (0.6–1.2)	1.0 (0.6–1.8)

p-value: Significant at ***<0.01

**<0.05

*<0.10.

^1^ Adjusting for clustering at EPI outreach center level.

The survey of high-risk group, conducted at CC, also show that those older respondents were more likely to accept all the three doses of COVID-19 compared to younger respondents aged 18–29 years. Similarly, respondents with higher level of education were more likely to get all the three doses of the vaccine compared to those with no education. Respondents who earned greater income, were more likely to accept the COVID-19 vaccine doses as well. This is depicted by respondents who earned an income equal to or greater than BDT 9000 per month- they were 3.1, 7.3, and 29.5 times likely to accept the consecutive doses of COVID-19 vaccine compared to respondents who earned less than BDT 3000 per month. However, among all our respondents, those suffering from chronic illness or had disability, were less likely to accept the vaccine doses compared to their healthy counterparts. An interesting finding of this survey is that transgenders were found to be 2.0 and 2.8 times likely to accept first two doses of the vaccine compared to male population of the survey. Notably, all the statistics reported from Table 5 are statistically significant.

## Discussion

This study was conducted with the aim to assess COVID-19 vaccination coverage as well as discern any disparities in vaccine uptake among selected vulnerable population of Bangladesh, i.e. rural population (both HTR and non-HTR areas) and urban population of high-risk group. Quantitative analysis depicted the presence of disparities within COVID-19 vaccine coverage. We have also presented the enablers and disablers for our study population in regards to receiving the vaccine. However, Bangladesh has successfully implemented the first and second doses of COVID-19 vaccination in almost all 16 districts of this study. Success were comparable across age, gender, education, economic and HTR and non-HTR groups. In contrast, we found vaccination coverage among CC-high risk groups (floating population, addicts, disabled and transgender) to be critically low with vaccine hesitancy being alarmingly high within the same groups. Furthermore, factors such as location of residence for rural areas (especially for rural HTR residents), level of education, financial stability, health condition were to have played a statistically significant role in the uptake of COVID-19 vaccine doses among our vulnerable study population.

Our analysis showed that coverage for the 1^st^ dose of COVID-19 vaccination was high and exceeded 90% among respondents from both rural HTR and non-HTR areas. We also found high coverage of COVID-19 vaccine among respondents in HTR rural areas and somewhat on par to their non-HTR counterparts. The high coverage in rural areas could be attributed to the fact that most rural respondents had received the COVID-19 vaccine from vaccination campaign and residents from HTR areas were not restricted by prior online registration but permitted to receive the vaccine as long as they turned up and were eligible age-wise. This is similar to results of a cross-sectional study which found high acceptance and uptake of COVID-19 vaccine in rural Bangladesh [18]. However, via our study, we observed a significant decrease in coverage of subsequent doses, especially the 3^rd^ dose. Results of multivariate regression analysis also showed that respondents from HTR were less likely to get vaccinated with the three doses of COVID-19 vaccine to their non-HTR counterparts. Notably, these findings have important policy implication since they imply the success of vaccination campaign as well as portray the need for further factors to be taken into consideration.

It is necessary for policies to be formulated that are specific to bringing the residents of HTR areas under vaccine coverage. Vaccination campaign need to be held in closer proximity of the population residing in HTR areas, preferably next to doorstep. Dissemination of vaccination information such as vaccination schedule, vaccination campaign location via miking needs to be considered as well given that most residents in rural HTR areas are homemakers with their husbands having migrated outside in search of work.

Vaccination coverage among urbanites of high-risk groups (floating population, addicts, disabled and transgender) was found to be critically low. The group had greater number of individuals who were chronically ill and/or disabled compared to their rural counterparts with a much greater number of individuals without institutional education. Disparities were especially conspicuous within the high-risk group. Across gender, female was found to be the least to have accepted the vaccine followed by men. Surprisingly, our study found a high vaccination coverage among the Trans respondents. This fascinating revelation could be attributed to the fact that they were motivated by their fellow mates and/or their leader (locally called ‘Guru Ma’) in addition to availability of vaccination at the doorstep. Female and individuals with lower education were less likely to receive the COVID-19 vaccine, a finding similar to another study which posited that female adolescents and/or adolescents who had lower education level were less willing to accept COVID-19 vaccine [19]. Furthermore, individuals with higher monthly income emerged as the highest sub-group that accepted the vaccine. This is similar to another study which showed that COVID-19 vaccination was lower among the low-income neighbourhoods in contrast to the high-income neighbourhood in Alberta [20]. Our findings also revealed that 92% of the high-risk group individuals did not have their vaccine cards and were the least likely to have access to healthcare and vaccine availability information. Overall, the urban individuals of high-risk group emerged to be the most deprived group of our study in terms of COVID-19 vaccine coverage with disparity spread across gender, educational status, and wealth status.

Notably, the disabled respondents both from rural and urban areas opted the least to accept vaccination with increasing number of doses. Such respondents, were found to be less likely to receive the vaccine doses compared to their healthy counterparts. It is suspected that such disinterest and lower coverage could be due to travel and transportation problem with a scarcity of vehicles well-suited to carry the disabled. This is somewhat similar to the findings of a study conducted in the USA where disabled respondents were less likely to have received one or more doses of the COVID-19 vaccine [21]. There is an increasing need for policymakers and policies to consider introducing low-cost disability-equipped vehicles or offering compensation for the disabled so that they can afford the travel cost to the vaccination campaign or centre.

Our study detected few motivating and discouraging factors across rural (HTR and non-HTR) and high-risk group respondents in regards to getting vaccinated. Among them, influence from others, information dissemination, vaccine hesitancy, fear of side-effects, and transport problem emerged to be the most prominent factors which can be addressed to improve the coverage. This is similar to another study conducted in rural Bangladesh which found the wish to protect others and the societal pressure to get vaccinated as enablers as well [18]. Based on the enablers and disablers, we recommend peer counseling via volunteers, school students and awareness raising activities through television or other media to increase the uptake of vaccine as well as apprise the general mass about vaccine schedule. Community engagement for raising awareness is always a useful method. To exemplify, a program can identify and use individuals who took the doses of vaccine to encourage their family members, friends and peers, neighbours, and other people of their community. Our findings show that most respondents were perceptive towards influence and encouragement from their family, peers and community members. Policies need to consider and encompass community engagement given how it played a vital role in motivating the respondents to get vaccinated with the COVID-19 vaccine doses. Distance and transportation affected both disabled and people who live far away from vaccine centers. Thus, policymakers may arrange for provision of extra vaccine spot or vaccination session targeting to this group of people when formulating policies for increasing immunization uptake as well as to tackle any future health crisis.

It is highly recommended that policymakers prioritize the high-risk group in regards to getting population vaccinated as well as during future health crisis. Our study has shown this category of vulnerable population to be the most deprived and vaccine hesitant. Individuals from this group could be provided risk group specific incentives (transport for disabled, monetary incentive to transgenders; food and medicine for drug user and floating people) and vaccination centers could be established with flexible schedule (morning/afternoon/evening sessions) so that they can get vaccine at their convenient time. Our findings imply that community engagement for the high-risk group individuals could be a successful step to abate their vaccine hesitancy and, an inference similar to another study finding that credited community engagement for the success of the implemented interventions and as a way to gain access to underprivileged population in selective districts of Bangladesh [22]. Registration prior to receiving vaccination was a prominent challenge for these individuals and so ‘single shot’ vaccine could be provided to them as GoB had arranged for floating drug users. Dissemination of information is especially important for the high-risk group as they have little to no access to healthcare information and are highly vaccine hesitant. Miking could be arranged at places where they are most noted to spend their time most such as the public points, especially informing them about special steps that GoB arranged specifically for the vulnerable such as vaccination at doorstep and ‘single shot’ vaccine.

### Strengths and limitations of the study

This study was conducted using a larger number of samples analysed via different kind of data collection techniques. The aim was to obtain concrete information about the topic which is the strength of this report. Furthermore, this study has a high external validity given our targeted study population and the statistical methods we employed. A few limitations of the study include possible response bias since one respondent at every household provided vaccination information of other household members in the survey in rural areas. However, we tried to validate the information from vaccination card when available which would limit this potential bias. Findings on COVID-19 vaccination coverage of this study cannot be compared with administrative coverage since this study obtained information only on all adult population of households whereas public administrative record include all individuals of eligible age for vaccination.

## Supporting information

S1 Table Socio demographic characteristics of the respondents. (DOCX)

S2 Table Place of vaccination for respondents who received at least a dose of vaccine by survey type. (DOCX)

S3 Table Status of vaccine card availability among respondents who received at least one dose of vaccine. (DOCX)

S4 Table Expenses incurred and paid by respondents who had at least one dose of vaccine. (DOCX)

S5 Table Reasons for getting vaccinated by respondents who received at least one dose of COVID-19 vaccine. (DOCX)

S1 Dataset Household survey. (ZIP)

S2 Dataset Survey with high-risk group. (ZIP)
