# Supplementary material for: Disparities in COVID-19 vaccine uptake among rural hard-to-reach population and urban high-risk groups of Bangladesh
Source: PLoS One. 2024 Apr 29;19(4):e0302056. doi: 10.1371/journal.pone.0302056 (PMC11057741; doi:10.1371/journal.pone.0302056)
Supplement: S1 Table — (DOCX) [file pone.0302056.s001.docx]

**S1 Table.** Socio demographic characteristics of the respondents

| **Characteristics** | **Rural: Household survey**  **(n=12,298)** | | **CC: High-risk group survey**  **(n=2,520)** | |
| --- | --- | --- | --- | --- |
|  | **Total** | **%** | **Total** | **%** |
| **Household location** |  |  |  |  |
| Hard-to-reach | 9274 | 75.4 | - | - |
| Non-hard-to-reach | 3024 | 24.6 | - | - |
| **Age** |  |  | **-** | **-** |
| 18-29 | 3115 | 25.3 | 401 | 15.9 |
| 30-39 | 3468 | 28.2 | 474 | 18.8 |
| 40-54 | 3491 | 28.4 | 677 | 26.9 |
| 55+ | 2224 | 18.1 | 968 | 38.4 |
| **Gender** |  |  |  |  |
| Male | 4151 | 33.8 | 1720 | 68.3 |
| Female | 8147 | 66.2 | 742 | 29.4 |
| **Transgender** | - | - | 58 | 2.3 |
| **Education status** |  |  |  |  |
| No education | 3121 | 25.4 | 1824 | 72.4 |
| Primary incomplete | 2065 | 16.8 | 506 | 20.1 |
| Primary completed | 2070 | 16.8 | 120 | 4.8 |
| Secondary incomplete | 2588 | 21.0 | 49 | 1.9 |
| Secondary completed or higher | 2454 | 20.0 | 21 | 0.8 |
| **Occupation** |  |  |  |  |
| Home maker | 7,260 | 59.0 | - | - |
| Farmer | 1,882 | 15.3 | - | - |
| Business | 764 | 6.2 | - | - |
| Day labourer | 924 | 7.5 | 61 | 2.4 |
| Student | 352 | 2.9 | - | - |
| Service | 437 | 3.6 | - | - |
| Beggar | - | - | 1711 | 67.9 |
| Not working^1^/Disabled | 595 | 4.8 | 563 | 22.3 |
| Porter | - | - | 170 | 6.7 |
| Others^2^ | 84 | 0.7 | 15 | 0.6 |
| **Wealth quintiles** |  |  |  |  |
| Lowest | 2467 | 20.1 | - | - |
| Second | 3146 | 25.6 | - | - |
| Middle | 2166 | 17.6 | - | - |
| Fourth | 2853 | 23.2 | - | - |
| Highest | 1666 | 13.5 | - | - |
| **Household income quintiles^3^** |  |  |  |  |
| Less than Tk. 3000 | - | - | 530 | 21.1 |
| Tk. 3000 - Tk. 5999 | - | - | 836 | 33.3 |
| Tk. 6000 - Tk. 8999 | - | - | 735 | 29.3 |
| Greater or equal Tk. 9000 | - | - | 408 | 16.3 |
| **Median Income (BDT)** | - | - | - | - |
| **Perceive health condition** |  |  |  |  |
| Healthy | 10979 | 89.3 | 1755 | 69.6 |
| Chronic illness | 1273 | 10.4 | 411 | 16.3 |
| Disabled | 46 | 0.4 | 354 | 14.0 |

^1^ For high-risk group it is coded as vagabond

^2^Others: Driver, Rickshaw/van puller, Skilled worker, Retired person, old allowance

^3^ at high-risk group survey,11 respondent coded as don't know

^4^ collected from client exit survey
