# Supplementary material for: Disparities in COVID-19 vaccine uptake among rural hard-to-reach population and urban high-risk groups of Bangladesh
Source: PLoS One. 2024 Apr 29;19(4):e0302056. doi: 10.1371/journal.pone.0302056 (PMC11057741; doi:10.1371/journal.pone.0302056)
Supplement: S2 Table — (DOCX) [file pone.0302056.s002.docx]

**S2 Table.** Place of vaccination for respondents who received at least a dose of vaccine by survey type

| **Vaccination center** | **Survey type (%)** | | | | | | |
| --- | --- | --- | --- | --- | --- | --- | --- |
|  | **Rural: Household survey** | | | **CC: High-risk group survey** | | |  |
|  | **1^st^ dose** | **2^nd^ dose** | **3^rd^ dose** | **1^st^ dose** | **2^nd^ dose** | **3^rd^ dose** |  |
|  | **(n=26,650)** | **(n=24,720)** | **(n=14,314)** | **(n=1,232)** | **(n=477)** | **(n=119)** |  |
| Vaccination Campaign | 56.9 | 58.5 | 69.3 | 72.4 | 45.9 | 29.3 |  |
| District Hospital | 4.7 | 4.4 | 3.6 | 4.6 | 8.0 | 5.7 |  |
| Upazila Health Complex | 37.3 | 36.0 | 26.3 | 17.1 | 34.4 | 54.7 |  |
| Medical College Hospital/ Specialized | - | - | - | 5.9 | 11.7 | 10.4 |  |
| Others | 1.1 | 1.1 | 0.8 | - | - | - |  |
