# Supplementary material for: Disparities in COVID-19 vaccine uptake among rural hard-to-reach population and urban high-risk groups of Bangladesh
Source: PLoS One. 2024 Apr 29;19(4):e0302056. doi: 10.1371/journal.pone.0302056 (PMC11057741; doi:10.1371/journal.pone.0302056)
Supplement: S3 Table — (DOCX) [file pone.0302056.s003.docx]

**S3 Table.** Status of vaccine card availability among respondents who received at least one dose of vaccine

| **Card shown** | **Survey type** | | | |
| --- | --- | --- | --- | --- |
|  | **Rural: Household survey** | | **CC: High-risk group survey** | |
|  | **n** | **%** | **n** | **%** |
| Yes | 8,842 | 77.0 | 98 | 7.9 |
| No | 2,640 | 23.0 | 1,134 | 92.1 |
| **Total** | **11,482** | **100** | **1,232** | **100** |
