# Supplementary material for: Disparities in COVID-19 vaccine uptake among rural hard-to-reach population and urban high-risk groups of Bangladesh
Source: PLoS One. 2024 Apr 29;19(4):e0302056. doi: 10.1371/journal.pone.0302056 (PMC11057741; doi:10.1371/journal.pone.0302056)
Supplement: S4 Table — (DOCX) [file pone.0302056.s004.docx]

**S4 Table.** Expenses incurred and paid by respondents who had at least one dose of vaccine

|  | **Survey type** | | | | | | | | |
| --- | --- | --- | --- | --- | --- | --- | --- | --- | --- |
| **Purpose** | **Rural: Household survey** | | | **CC: High-risk group survey** | | | **Client exit survey** | | |
|  | **n=9,369** | | | **n=227** | | | **n=327** | | |
|  | Total | % | Median cost^*^ | Total | % | Median  cost^*^ | Total | % | Median cost^*^ |
| Registration/card | 8979 | 95.8 | 30 | 187 | 82.4 | 30 | 139 | 42.5 | 30 |
| Travel | 6673 | 71.2 | 120 | 185 | 81.5 | 120 | 312 | 95.4 | 60 |
| Others | 327 | 3.5 | 55 | 5 | 2.2 | 20 | 22 | 6.7 | 15 |

^*^Cost in BDT
