# Supplementary material for: Disparities in COVID-19 vaccine uptake among rural hard-to-reach population and urban high-risk groups of Bangladesh
Source: PLoS One. 2024 Apr 29;19(4):e0302056. doi: 10.1371/journal.pone.0302056 (PMC11057741; doi:10.1371/journal.pone.0302056)
Supplement: S5 Table — (DOCX) [file pone.0302056.s005.docx]

**S5 Table.** Reasons for getting vaccinated by respondents who received at least one dose of COVID-19 vaccine

| **Reasons for getting vaccinated*** | **Survey type (%)** | |
| --- | --- | --- |
|  | **Rural: Household survey** | **CC: High-risk group survey** |
|  | **(n=26,650)** | **(n=1,232)** |
| To protect ownself from coronavirus | 90.4 | 80.4 |
| To protect family | 36.8 | 5.1 |
| Following others | 17.2 | 48.1 |
| Advised by health care providers | 16.2 | 17.2 |
| Advised by family members | 16.0 | 0.7 |
| Advised by relatives/ friends | 7.9 | 4.2 |
| Others | 12.1 | 5 |

**Multiple responses*
